# Ethically acceptable consent approaches to adolescent research in South Africa

**DOI:** 10.4102/sajhivmed.v23i1.1385

**Published:** 2022-09-05

**Authors:** Marian Loveday, Ameena Goga, Ames Dhai, Melodie Labuschaigne, Theresa Roussouw, Theresa Burgess, Ann Strode, Melissa Wallace, Marc Blockman, Brodie Daniels, Elizabeth Spooner, Linda-Gail Bekker

**Affiliations:** 1 HIV and other Infectious Diseases Research Unit (HIDRU), South African Medical Research Centre, Durban, South Africa; 2 Centre for Health Systems Research and Development, University of the Free State, Bloemfontein, South Africa; 3 CAPRISA-MRC HIV-TB Pathogenesis and Treatment Research Unit, University of KwaZulu-Natal, Durban, South Africa; 4 Department Paediatrics, University of Pretoria, Pretoria, South Africa; 5 School of Clinical Medicine, Faculty of Health Sciences, University of the Witwatersrand, Johannesburg, South Africa; 6 Department of Jurisprudence, School of Law, University of South Africa, Pretoria, South Africa; 7 Department of Immunology, University of Pretoria, Pretoria, South Africa; 8 UP/SAMRC Research Centre for Maternal, Fetal, Newborn and Child Health Care Strategies, University of Pretoria, Pretoria, South Africa; 9 Division of Physiotherapy, University of Cape Town, Cape Town, South Africa; 10 Centre for Medical Ethics and Law, Stellenbosch University, Stellenbosch, South Africa; 11 School of Law, College of Law and Management Sciences, University of KwaZulu-Natal, Pietermaritzburg, South Africa; 12 HIV/AIDS Vaccines Ethics Group, School of Applied Human Sciences, College of Humanities, University of KwaZulu-Natal, Pietermaritzburg, South Africa; 13 The Desmond Tutu HIV Centre, University of Cape Town, Cape Town, South Africa; 14 Division of Clinical Pharmacology, University of Cape Town, Cape Town, South Africa

**Keywords:** adolescents, parental waiver of consent, ethics, conflicting legal regulations and statutes, research exclusion

## Abstract

**Background:**

Adolescents are a unique population with significant unmet health needs. They are often excluded from research that may benefit them as they are perceived as vulnerable and needing protection from research participation. For Research Ethics Committees, conflicting positions in statutes, regulations and ethical guidelines about who provides informed consent for adolescent involvement in health research can be a significant barrier to approving adolescent research. For researchers, the requirement for parental/guardian proxy consent or prolonged approval processes may potentially result in the exclusion of those adolescents most vulnerable and at risk, particularly if issues such as gender-based violence, gender identity, sexuality and sexual practices are in question.

**Objectives:**

To describe the challenges to adolescent research and suggest strategies to address these.

**Method:**

We consider the legal and ethical framework in South Africa regarding the consenting age for adolescents in research, outline the challenges and, using examples of best practices, suggest strategies to address the current conundrum.

**Results:**

We suggest three principles to guide Research Ethics Committees on their approach to reviewing health research involving adolescents. Strategies to develop ethically acceptable approaches to adolescent research and consent processes are described, which include community involvement. We elaborate on examples of nuanced approaches to adolescent research.

**Conclusion:**

The inclusion of adolescents in research is critical in informing appropriate and effective health services for this vulnerable population, whilst providing an opportunity to link them into care and services where relevant.

## Introduction

Adolescence, defined by the World Health Organization and Statistics South Africa as those 10–19 years old, is a transitional stage from child- to adulthood.^1,2^ This life period is one of orientation and discovery as questions of independence, identity and a sense of self emerge. It is a time of heightened vulnerability as choices about future, friendship, sexuality, gender identity, and substance use are negotiated. With an evolving capacity to make sound decisions and participate in promoting their own welfare, the needs and vulnerabilities of pre- and early (10–13-years-old), middle (14–16-years-old) and late adolescents (17–19-years-old) vary.^3^

Globally 1.2 billion adolescents make up 16% of the world’s population, with 90% living in developing countries, where they make up 23% of the population.^4^ The vulnerabilities and needs of adolescents and adolescent girls, in particular, often remain unaddressed. Large numbers of unsafe abortions and a high risk of pregnancy-related mortality occur, with 11% of the global births in girls occurring before their 18th birthday.^5,6,7^ Health services for adolescents are increasingly recognised as a priority in low- and middle-income countries,^8^ as it is during adolescence that the foundations for adult health and well-being are laid, underpinning the health and well-being of subsequent generations.

The unique health needs of adolescents are seldom addressed, a situation which is further exacerbated by their exclusion from research. Exclusions may occur as adolescents require a different and unique approach to that of adults, perceived to be vulnerable, needing protection from possible risks of research involvement, and perceived to be incapable of autonomous consenting with good understanding. Ironically, although ethical considerations regarding adolescent involvement should be a balancing act between protection from research harm and inclusion in research that may bring benefit, these ethical issues have themselves become a barrier to adolescent inclusion in research. In South Africa, conflicting positions in statutes, regulations, and guidance on who provides informed consent for adolescent involvement in health-related research has become a significant challenge and consequently a barrier. This situation is exacerbated by misunderstandings of adolescents’ cognitive abilities and capacity to provide informed consent, valuing of protection over inclusion, institutional self-protection by Research Ethics Committees (RECs) and antiquated attitudes about adolescents not holding equal rights to adults.^9,10^

This situation is no longer tenable, as RECs find themselves increasingly in unenviable positions and researchers are frustrated.^10^ Moreover, the frequent exclusion of adolescents from research is compromising their health and well-being.^11,12^ In this article, we consider the legal and ethical framework in South Africa with regard to the consenting age for adolescents in research. We outline the challenges related to differing requirements of legislation and guidelines and, using examples of best practices, suggest strategies to address the current conundrum.

## Current legal and ethical situation in South Africa

Health research is regulated by legal norms set out in the *National Health Act* (NHA).^13^ These norms must be read in conjunction with other pieces of legislation which set standards relating to child and adolescent health. In addition, and overlapping the law, are ethical obligations to health research.

### *National Health Act* 61 of 2003 and regulations relating to research with human participants

Research involving minors is governed by section 71 of the NHA,^13^ which requires mandatory consent from the parent or guardian. If the minor has the ability to understand, he or she consents (not assents), together with the parents or guardian.^13^ The significance of a minor’s consent and not assent is borne out by the discussion below which underscores capacity to consent rather than an age-dependent consent framework. There are no permissible exceptions to parental/guardian consent with respect to proxy consent and waiver of consent.

The NHA is complemented by two provisions from the 2014 Regulations that relate to research with human participants^14^: (1) Regulation 4.1 states that research with minors should only take place when adults are *not* appropriate participants for the research; if the research poses no more than a minimal risk to the minor; if the research poses more than a minimal risk, that it has a direct benefit to the minor; or where the research poses more than minimal risk and has no direct benefit to the minor, it may contribute to generalisable knowledge of the health condition being studied; (2) Regulation 2 establishes the 2015 Department of Health Guidelines on Ethics in Health Research as the mandatory minimum benchmark for health research, thereby recognising the legal force of the Guidelines. It stipulates that health research be undertaken with ‘appropriate consent processes’, but gives no guidance on how the inconsistency in consent approaches for minors’ participation in research in the NHA, Regulations and Department of Health Ethical Guidelines should be addressed.

### *Children’s Act* 38 of 2005 and the choice on the Termination of Pregnancy Act 92 of 1996

The *Children’s Act*, in contrast to the NHA, recognises the decision-making autonomy of children as human rights bearers, based on their evolving maturity and mental capacity to participate in decisions that affect them.^15,16^ In other words: children, as persons and constitutional rights bearers with an evolving capacity for individual autonomy, have the right to make their own decisions regarding matters that affect them, provided they display sufficient maturity. Examples of context-specific interventions where children’s consent is recognised in the Act include, amongst others, access to contraceptives, medical treatment and HIV testing. Similarly, the *Choice on the Termination of Pregnancy Act* provides that children do not need parental consent for termination of an early pregnancy.^17^

As the *Children’s Act* does not regulate research involving minors it does not offer direct guidance in resolving the different consent approaches for minors. However, it is counter-intuitive to allow children to independently access services that impact their sexual, general, and mental health, but deny them the opportunity to participate in research which may benefit their health individually and collectively, on grounds of diminished capacity and autonomy.

### The 2015 Department of Health Guidelines on ethics in health research^18^

The Department of Health Ethical Guidelines state that minors should participate in research only if the research is relevant to them, cannot be achieved without their involvement, and is in their best interests with minimal or negligible risk of harm. These guidelines are to some extent aligned with the NHA on minors’ consent for research, but deviate from the NHA’s *mandatory* parental or legal guardian consent requirement allowing for REC discretion when the participation of minors is considered.^11^ They provide that, in specific instances: (1) a parental substitute may be appointed to provide consent; and (2) the minor themselves can consent independently to research participation. For example, for studies about sexual activity or substance abuse it may be ethically justifiable and appropriate for mature minors (e.g. 16 years and older) to independently consent to research participation as the involvement of parents/guardians may potentially result in social harm to the minor or may result in the failure of adolescents to participate because of their fear of disclosure. The guidelines recommend prior engagement with community role players to justify minors’ independent consent and that RECs may grant a waiver of the requirement of written parental permission. In these cases, the process should be carefully documented.

### The current conundrum: The 2015 ethical guidelines versus the *National Health Act* and regulations

As Regulation 2 of the 2014 Regulations has endorsed the Ethical Guidelines as the minimum legal standard for research with human participants, section 71 of the NHA, the 2014 Regulations and the Ethical Guidelines are all legally binding. However, as the legislation and guidelines provide conflicting stipulations with respect to the consent process for research, we suggest four principles to guide RECs on which legal instrument should prevail in reviewing health research involving minors.

Firstly, where legal provisions on a specific issue may lead to conflicting interpretations, an attempt should be made to harmonise the interpretations in a manner that is aligned with the values enshrined in the Constitution or the Bill of Rights.^19,20^ The Constitution endorses the Bill of Rights as the cornerstone of democracy in South Africa and furthermore directs courts to promote the object, purport and spirit of the Bill of Rights.^20^ Consequently, an interpretation of the NHA, regulations and the *Children’s Act* should strive to give effect to and promote the aims and values of the Constitution and the Bill of Rights, which in the case of children’s consent would favour an interpretation that acknowledges the evolving decisional autonomy of children. The *Children’s Act*’s recognition of children’s decisional capacity is more attuned to the Bill of Rights and its interpretation is hence to be preferred above that of the NHA. Secondly, if there is a conflict in the provisions of two statutes dealing with the same subject (e.g. children’s consent), the general rule in South African law is that the later provision should be followed.^21,22^ Not only was the *Children’s Act* promulgated after the NHA, but it gives better effect to children’s rights in general, as well as to their autonomy. Thirdly, since rules of statutory interpretation provide that any conflict between a prevailing act and any of its regulations be resolved in favour of the prevailing act,^23^ this would technically favour the NHA’s interpretation over the interpretation in its (2014) Regulations. However, as pointed out above, since the NHA’s interpretation is inconsistent with the Constitution and the Bill of Rights with regard to children’s autonomy, the recommended legal position regarding children’s consent to research should be the position captured in the 2015 Guidelines, whose interpretation supports and promotes the autonomy of children. Fourthly, the Constitution mandates the consideration of international law in the interpretation of the Bill of Rights, which includes international agreements such as the United Nations Convention of the Rights of the Child signed by South Africa in 1993, requiring an interpretation of national legislation that is aligned and compliant with international law.^20,24^

The United Nations Convention of the Rights of the Child recognises children as rights holders, draws attention to their protection and provision of rights, the obligation to consider their best interests and their evolving capacities to make sound decisions and participate in promoting their own welfare. Although it does not refer specifically to research, its articles are flexible enough to address participation in research. Numerous other international documents have also addressed the issue of minors and research and the main themes emerging from these documents are: (1) children are people in their own right and have a right to have a say and be heard, including in the context of well-planned, ethical research; (2) involvement of children in any kind of research should take place in partnership with caring, skilled adults who provide appropriate support and guidance; (3) research should focus on understanding and improving children and adolescents’ lives and circumstances; and (4) a participatory approach in which children are included in the research process should be promoted.^25,26,27,28,29,30^

The ongoing legal uncertainty as to whether the NHA imposes a strict mandatory parental consent approach or whether it can be interpreted in a purposive manner to align it with the norms in the Regulations and national Ethical Guidelines remains contentious. This article does not address this issue as authors have written about it elsewhere.^10^ Instead, it proceeds on the basis that children have a fundamental right to benefit from scientific advances and, to fulfil this right, discussion and debate is needed with RECs and researchers on how to do this in an ethical and principled manner.

## Recommendations/possible ways forward

### Getting the framework straight

**FIGURE 1:**
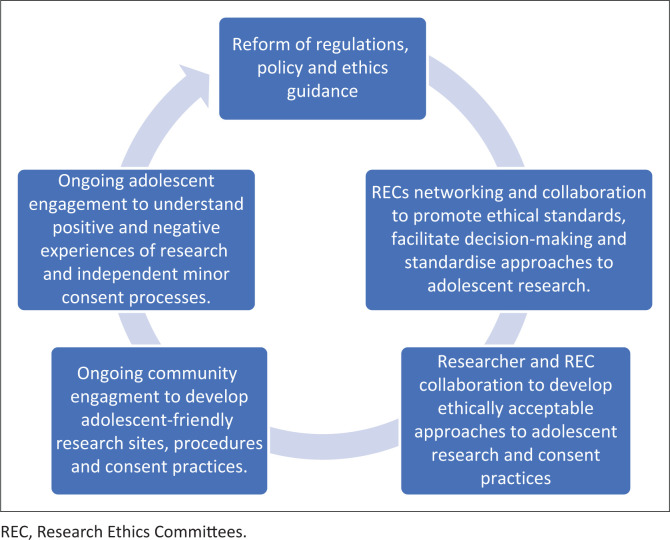
Developing an appropriate framework.

The inclusion of adolescents in research is needed to advance their health. The strategies detailed below to develop ethically acceptable approaches to adolescent research and consent practices are illustrated in Figure 1.

- **Reform:** Regulatory, policy and ethical guidance reform are needed to facilitate research involving adolescents. A minimum is closer alignment between the legal and ethical requirements.
- **Collaboration between RECs:** Research Ethics Committees need to collaborate and work together to promote ethical standards, facilitate decision-making around ethical challenges, and through a consensus derived approach standardise approaches to adolescent research within an enabling framework. The generation of a publicly available ‘ethics repository’ of resources to support all stakeholders in adolescent research would develop this capacity in South Africa.
- **Dialogue and feedback between researchers and RECs:** To develop a common understanding, opportunities for feedback and dialogue between researchers and RECs need to be established. This would provide an opportunity to inform RECs of the positive and negative experiences of independent minor consent processes if parental consent is waived. This feedback may be required as part of the approval process to ensure real-time monitoring, accountability, and learning. Additionally, reporting back ongoing research findings and adolescent-informed community stakeholder experiences and recommendations would facilitate the amendment of consent approaches to protect the best interests of adolescent participants in an iterative fashion.
- **Community engagement:** This is addressed in the section below and should be operationalised.
- **Adolescent-informed engagement:** Adolescent-informed engagement strategies are needed to guide research involving adolescents from different sociocultural backgrounds with divergent views of when parental consent is acceptable or necessary. Consent procedures may need to be tailored to accommodate the diverse needs and preferences of adolescent participants.

### How to go about ethically acceptable research with minors

**FIGURE 2:**
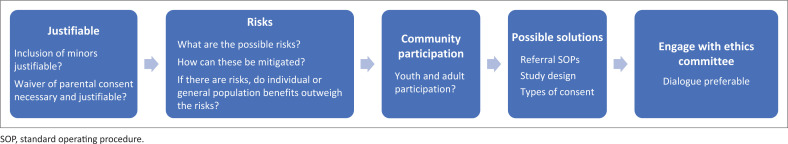
Steps to initiating ethically acceptable research with minors.

Before embarking on research with minors, researchers need to ensure they are familiar with the complexities of parental consent and how these are addressed in the NHA, subsequent regulations and the Ethical Guidelines. These will provide guidance in addressing the issues that need to be considered (Figure 2). Firstly, is the inclusion of minors necessary and justifiable? And, if a waiver of parental consent is being applied for, is this necessary and justifiable? Secondly, when designing the research study, the level of risk must be assessed, as the Ethical Guidelines stipulate that independent minor consent is only appropriate for minimal risk research.^18^ Thirdly, how can both youths and adults be engaged to ensure their input and participation throughout the study process? Trained Community Advisory Boards (CABs) that involve youth/minors may achieve this, but in their absence, can community participation be ensured? Fourthly, standard operating procedure documents for serious health issues need to be developed, detailing referral procedures to support structures, for instance, for minors diagnosed with sexually transmitted infections including HIV and those who become pregnant, together with mandatory reporting procedures for abuse and neglect of minors, and under-age sexual activity. Fifthly, timely and continual dialogue with RECs to protect adolescents whilst facilitating their participation in research.

### The importance of community engagement in adolescent studies

One of the requirements for independent consent by minors in the Ethical Guidelines is prior engagement with ‘participating community role players’ with tangible evidence of such engagement.^18^ Based on the principle that individuals are interrelated and decisions are made within a social context, the purpose of community engagement is to assess whether parents and the wider community are supportive of minors’ involvement in the research without explicit individual-level parental consent. Community engagement should never be tokenistic or seen as an ancillary component of the research process; it should be authentic, objective and effective. Through the exchange of research ideas and community opinions throughout the research process, new and mutually beneficial connections will develop, benefitting the research agenda.^31,32^

Community engagement is challenging, as even a definition of community is contentious. Five defining characteristics of communities have been suggested: locus, sharing, joint action, social ties, and diversity; but as communities are increasingly heterogeneous and dispersed, community engagement should involve different strategies and be as inclusive as possible.^33,34^ For adolescent research, stakeholders could potentially include parents, foster parents, guardians, adolescents of the relevant age range including transgender people and sexual minorities, representatives of child-headed households, teachers, principals and social workers. In addition, key decision-makers (district level government and non-governmental stakeholders and community leaders) may need to be included to facilitate the eventual translation of research findings into policies and practices. Researchers need to be aware of the socio-economic context and power relations which result in competing interests, priorities and structural coercion threatening the legitimacy and ethical conduct of the research.^35,36^

Many documents describe how to ensure successful community engagement.^27,31^ Most guidance is based on a CAB model, where CABs create a forum through which questions, concerns, and community needs can be raised; the feasibility of protocols determined; bidirectional sharing of information to facilitate understanding of the research and the community channelled; culturally acceptable recruitment strategies identified; and, ultimately, research support generated among the broader community. The Tygerberg research Ubuntu-inspired community engagement (TRUCE) model (Box 1) details eight steps for successful community engagement.^34^ Staff at research sites do well to pay attention to community engagement and establish representative CABs before any research protocols are embarked upon. Ensuring CABs are well constituted and continue to function autonomously and effectively requires ongoing time, resources, and expertise.

BOX 1 Eight steps for successful community engagement.

- Identify potential communities through social mapping.
- Establish the scope of community engagement.
- Approach communities early.
- Co-create strategies for community engagement.
- Acknowledge and respect co-ownership of knowledge.
- Recruit and engage communities at each stage of research.
- Evaluate and adapt community engagement strategies.
- Discuss and disseminate results after the research is completed.

*Source:* Moodley K, Beyer C. Tygerberg research Ubuntu-inspired community engagement model: Integrating community engagement into genomic biobanking. Biopreserv Biobank. 2019;17(6):613–624. https://doi.org/10.1089/bio.2018.0136

Research protocols should include a description of community engagement activities, the roles, responsibilities and skills of various parties, training needs, how feedback will be given to the communities, together with innovative ways of involving the community during the dissemination of information, including publications and conference presentations.

### Best practice examples of waivers of consent in adolescent research

One of the first South African studies involving adolescents in HIV vaccine research highlighted requiring parental/guardian consent as a barrier to the enrolment of high-risk adolescents; careful consideration of the consent approach is advisable.^37^

The Desmond Tutu HIV Centre has been conducting clinical research with adolescents since 2003 and has developed a nuanced approach to consent, depending on the nature of the study and age of the adolescents participating in the research. A ‘one-size-fits-all’ approach is not possible, and each study requires an individualised, context-specific exercise.^38^ The design of these approaches is developed in consultation with well-established adult and youth CABs including representative ages, populations and stakeholders’ input, guidance from ethical and legal experts to ensure protective ‘safety nets’ are in place, and open and ongoing communication with RECs. Depending on the study, adolescents are given the option to involve a trusted adult or parent/guardian, and a parental proxy consent form provided. Some examples of these studies, together with the different reasons for waivers of parental consent, are described in Table 1.

**TABLE 1:** Examples of adolescent studies undertaken at the Desmond Tutu HIV Centre, together with the rationale for a waiver of parental consent.

Adolescent studies	Rationale for a waiver of parental/guardian consent	Acceptable ICF solution
**The goals for girls study**, a randomised controlled trial to evaluate a sport-based sexual and reproductive health programme implemented among 14–17-year-old girls across 40 secondary schools in Cape Town.	A non-therapeutic study of low to minimal risk for participants. Requiring parental consent could be a barrier to accessing much-needed services for these young women.	All participants were minors and at school. School leadership and a youth advisory group were involved in the consent discussion. Parental consent was waived, but participants could elect to have parental consultation and consent.
**The BUDDY (Bidirectional, Upbeat communication and Differentiated, Distanced care for Young people) study** recruited 13–24-year-olds living with HIV on ART to examine a novel remote service delivery model to facilitate engagement in HIV care during the COVID-19 pandemic. A cohort of HIV-negative young people was included and, across both groups, the impact of COVID-19 lockdowns, with a focus on gender-based violence, was investigated.	Parental consent would require disclosure of HIV status. In addition, as the study was exploring experiences of gender-based violence and violence, parents or others in the adolescents’ households might be implicated. Requiring parental consent could bias the sample, place adolescents at risk, and exclude those most vulnerable (adolescents living with HIV without disclosure to parent/legal guardian or living in a household with violence). Participation would facilitate access to care, treatment, and social support.	Parental consent was waived in minor participants. Regardless of age, participants could elect to have parent/legal guardian consent, and/or have a trusted adult (of the participant’s choice) review the consent form and assist in their decision to participate in the study.
**The CHAPPS (Combined HIV Adolescent PrEP and Prevention) Study** aimed at examining the acceptability and feasibility of providing daily and on-demand PrEP to HIV-negative, sexually active adolescent boys and girls, aged 13–24 years, through focus groups, interviews and surveys.	Seeking parental consent would require disclosure of sexual activity to parents/legal guardians, which may have negative consequences for adolescents. This was a behavioural study without the actual provision of PrEP.	Parental consent was waived. Participants could optionally seek parental consent.
**Pluspills:** This study of (F/TDF) oral PrEP in adolescent boys and girls aged 15–19 years was conducted in 2015–16 when PrEP, although licensed in adults, was still considered experimental for prevention in minors (used extensively in treatment).^39^	PrEP use was suggestive of sexual activity and the protocol required sexual debut for eligibility. This was weighed up against the fact that, at this time, PrEP was still experimental at study initiation. The research team opted for adolescent participation with parental knowledge and involvement. Youth CAB consulted and was welcoming of research. Adult CAB also welcomed research, but had many questions about this novel intervention.	Parental consent was sought. All 150 participants were recruited including a significant number of minors. The requirement for parental involvement may have impacted enrolment of sexually active adolescents < 18 years who were not ready to disclose to parents.

ICF, Informed Consent Form; ART, Antiretroviral Therapy; PrEP, Pre-exposure prophylaxis; F/TDF, emtricitabine/tenofovir disoproxil fumarate; CAB, Community Advisory Boards.

## Conclusion

In early November 2021, Statistics South Africa released a report on pregnancy in South Africa. There were over 1 million babies born in 2020, of which 34 587 were born to girls ≤ 17 years old and 600 to girls aged 9 and 10. Eighty per cent of HIV infections occurring in adolescents in sub-Saharan Africa occur in girls 15–19 years of age, where the prevalence of HIV in adolescent girls and young women is four times higher than in young men, with one-third screening positive for mental health and substance abuse problems.^40,41,42,43^ In addition, a quarter of sexually active adolescent girls contract a curable sexually transmitted infection annually.^44^

We as a country are failing to protect our girl children and adolescents and every possible measure must be taken to address this situation as a matter of urgency. Our efforts are more likely to be effective if evidence-based interventions are employed. Firstly, the NHA should be amended to take into consideration the United Nations Convention of the Rights of the Child’s provisos on the rights of children and their evolving capacities to make informed decisions. Secondly, the National Heath Research Ethics Council needs to work together with RECs to standardise approaches to adolescent research to ensure adolescents are not excluded from research that will benefit them. Thirdly, researchers and RECs need to work together so that a more informed position based on the experiences of parental waivers of consent is developed. Essentially, what is required is to balance child protection with the facilitation of ethical child and adolescent research. In the meantime, researchers, communities, and ethics committees must find ways to include adolescents safely and ethically in relevant and justifiable research.

By investing in adolescent health, we are investing in the health and productivity of future generations. The inclusion of adolescents in research is critical in informing appropriate and effective health services for this vulnerable population whilst providing an opportunity to link them into care and services where relevant. While it is critically important to acknowledge adults in gatekeeping roles for protecting adolescents from potential harm, it is equally important to respect adolescents’ right to privacy and their evolving capacities to take autonomous responsibility for their health and health decisions.
